# Intracholecystic administration of indocyanine green for fluorescent cholangiography during laparoscopic cholecystectomy—A two-case report

**DOI:** 10.1016/j.ijscr.2020.02.054

**Published:** 2020-02-28

**Authors:** Man-Ling Jao, Yen-Yu Wang, Hon Phin Wong, Sayali Bachhav, Kai-Che Liu

**Affiliations:** a Department of Surgery, Show Chwan Memorial Hospital, Changhua, Taiwan; b IRCAD/AITS-Asian Institute of TeleSurgery, Chang Bing Show Chwan Hospital, Changhua, Taiwan

**Keywords:** PTGBD, percutaneous trans-hepatic gallbladder drainage, ICG, indocyanine green, LC, laparoscopic cholecystectomy, CVS, critical view of safety, NIRF, near-infrared fluorescent cholangiography, IV, intra-venous, Fluorescent cholangiography, Intracystic administration, Indocyanine green, Laparoscopic cholecystectomy, Intracystic ICG

## Abstract

**Introduction:**

The utility of intracystic administration of indocyanine green for near-infrared fluorescent cholangiography in acute calculous cholecystitis initially treated with percutaneous transhepatic gallbladder drainage (PTGBD) was described in this report.

**Presentation of case:**

Two cases who underwent near-infrared fluorescent cholangiography guided interval laparoscopic cholecystectomy two weeks post-PTGBD were studied retrospectively. Both patients were diagnosed with moderate acute calculous cholecystitis based on diagnostic criteria of the Tokyo guidelines. Two routes of indocyanine green administration were utilized during surgery, first through direct intracystic administration through PTGBD tube (5 ml of 12.5 mg ICG) to achieve critical view of safety and then intravenous administration (1 ml of 2.5 mg ICG) to visualize cystic artery.

**Discussion:**

Both patients had critical view of safety visualized clearly with ICG with the operation time of 84 and 125 min in cases 1 and 2, respectively without any intra or postoperative complications.

**Conclusion:**

In comparison with intravenous ICG administration, trans-PTGBD ICG route can provide better signal-to-noise ratio by avoiding hepatic fluorescence and thus increasing the bile duct to liver contrast. However, ICG may enter the lymphatic system through necrotic and inflammatory gallbladder mucosa, of which lymph spillage during gallbladder dissection can obscure the fluorescent view.

## Highlights

• It is difficult to visualize extra-hepatic biliary anatomy clearly because of long-presence of ICG in liver when administered intravenously.
• Intracholecystic ICG injection illuminates extra-hepatic biliary tree preferentially thus reducing background hepatic noise.
• Surgeons can experience more satisfaction with the use of fluorescent cholangiography during laparoscopic cholecystectomy when the intracystic route of ICG administration is utilized.

## 1 Introduction

Laparoscopic cholecystectomy (LC) is one of the most common abdominal laparoscopic surgery performed globally. Even though LC is regarded as more safe and time-saving procedure compared to open cholecystectomy, it comes with increased incidence of bile duct injury and related morbidity because of misidentification of cystic duct due to its peculiar operative environment [1]. To mitigate the challenge, the methods to clearly visualize extra-hepatic biliary anatomy during surgery have been advocated.

Critical view of safety (CVS) is a ductal identification technique by ensuring that only two structures enter the gall bladder, cystic duct and cystic artery, and thus helps to avoid bile duct injuries during laparoscopic cholecystectomy [2]. Near-infrared fluorescence imaging (NIRF) provided real-time intraoperative mapping of the biliary tree and was proven to be effective and feasible to visualize the extrahepatic biliary system [3]. Comparing intraoperative cholangiography and NIRF, a systematic review reported similar outcomes, in terms of achieving CVS [3,4].

**Fig. 1:**
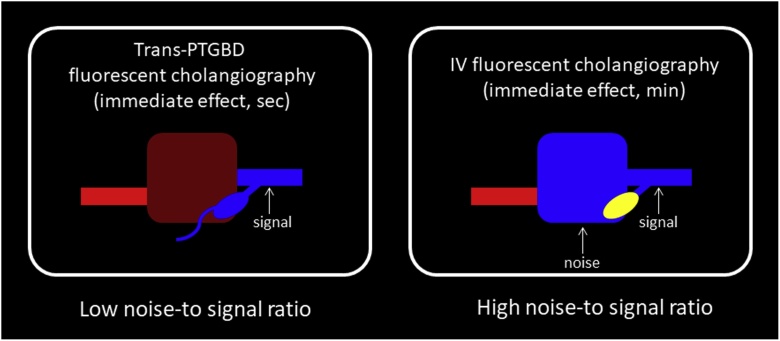
Comparing the IV and trans-PTGBD routes, liver parenchyma is illuminated after IV ICG injection resulting in high noise-to-signal ratio. The trans-PTGBD route displays a low noise-to signal ratio which highlights the extrabiliary system.

During NIRF indocyanine green (ICG) which is a FDA approved safe, easily available and stable fluorescent dye is injected intravenously (IV) where it binds to proteins and gets excreted into bile subsequently. Many studies [[5], [6], [7]] have administered ICG 15–120 min prior to anaesthesia to achieve optimum visibility of biliary tree. However, the time interval for IV administration should take into consideration high noise-to-signal ratio with background hepatic illumination due to prolonged ICG fluorescence in humans. The commonly applied intravenous route can result in intraoperative background liver fluorescent signal, which would disturb the visualization of biliary structures (Fig. 1) [7,8]. Verbeek et al. [9] proposed that longer the interval between ICG administration and surgery, lower is the noise-to-signal ratio in NIRF cholangiography, and thus supported ICG administration 24 h prior to the surgery to reduce background liver enhancement and increase bile duct to liver contrast.

Liu et al. [10] recently proposed the alternate route for ICG administration directly into gallbladder either from percutaneous transhepatic gallbladder drainage site or intraoperative cystic injection followed by a purse-string suture. Direct intracystic administration would be beneficial to achieve lower noise-to-signal ratio as the ICG would be circulated through biliary structures preferentially. To investigate the utility of this technique further, current study demonstrated the application of NIRF LC in two patients with moderate acute cholecystitis with ICG administration via matured percutaneous transhepatic gallbladder drain. The study has been reported according to the SCARE guidelines [11].

## 2 Presentation of case

Two male patients, over 70 years old with the diagnosis of moderate acute cholecystitis according to Tokyo guideline, 2013 and undergone interval LC [12,13] after percutaneous transhepatic gallbladder drainage (PTGBD) in urgent settings at our memorial hospital during May 2015 to October 2016 were studied retrospectively.

### 2.1 Case report 1

An 84-year old male patient with history of hypertension and type-II diabetes mellitus presented with right upper quadrant abdominal pain associated with intermittent fever for 3 days to emergency department after having fatty food. On physical examination Murphy’s sign was positive with no other abnormalities. Patient’s blood report showed abnormal hepatobiliary profile with elevated leucocyte count. Abdominal CT confirmed the diagnosis of moderate acute cholecystitis with cholelithiasis and cystic duct stone. PTGBD was performed to manage acute cholecystitis in urgent settings in accordance with the management criteria of Tokyo Guidelines 18 (TG18) based on patient’s probable surgical risks and complications [13]. Patient was scheduled for interval LC along with broad-spectrum antibiotics treatment prior to surgery. After two weeks, on T-tube cholangiography there was no presence of CBD stone or other biliary pathology. NIRF-guided LC was performed.

### 2.2 Case report 2

A 73-year old male patient with history of hypertension, type-II diabetes mellitus and benign prostatic hyperplasia was diagnosed with moderate acute cholecystitis with gall bladder stone. Abnormal renal index and hepatobiliary profile was noted on blood reports. T-tube cholangiography was suggestive of gall stones and ruled out presence of CBD stone. Considering the multi-comorbid conditions, patient was discharged with percutaneous transhepatic gall bladder drain in-situ to manage acute cholecystitis [13] and was scheduled two weeks later for elective LC. On physical examination, the yellowish discharge was observed at drainage site. Antibiotics and analgesic therapy was given before surgery. After successful pre-operative assessment, NIRF-guided LC was performed.

### 2.3 NIRF-guided laparoscopic cholecystectomy

In both cases, the surgery was performed with standard three port approach by same team of surgeons having 8 years of surgical experience. ICG (25 mg vials; Diagnogreen; Daiichi Sankyo Co, Tokyo, Japan) was resuspended in 10 ml of sterile water for injection to yield a 2.5-mg/ml stock solution. Of this stock solution 5 ml, corresponding to the dose of 12.5 mg, was administered intracystically as a bolus via two-week fistularized PTGBD right after all trocars were inserted. Laparoscopic images were acquired using the Image 1 High Definition fluorescence laparoscope (Karl Storz Endoscopes, Germany).

**Fig. 2:**
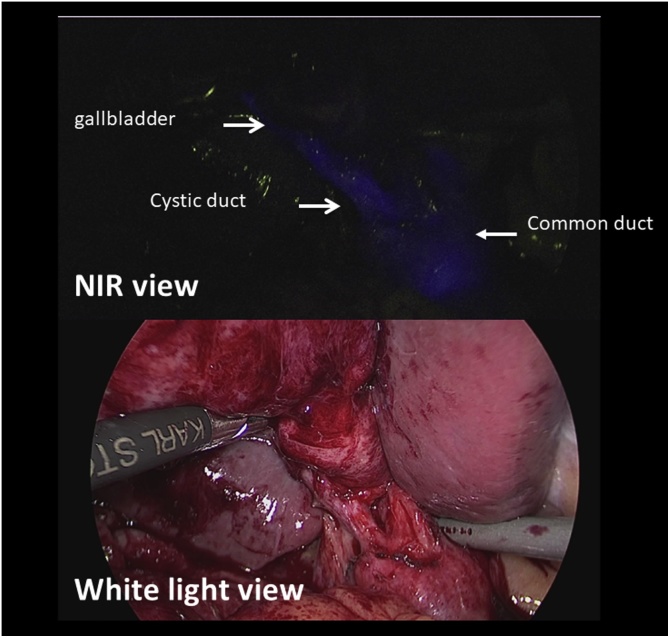
Angulated cystobiliary junction of case 1 under NIRF after trans-PTGBD ICG injection. The identical view under white light.

**Fig. 3:**
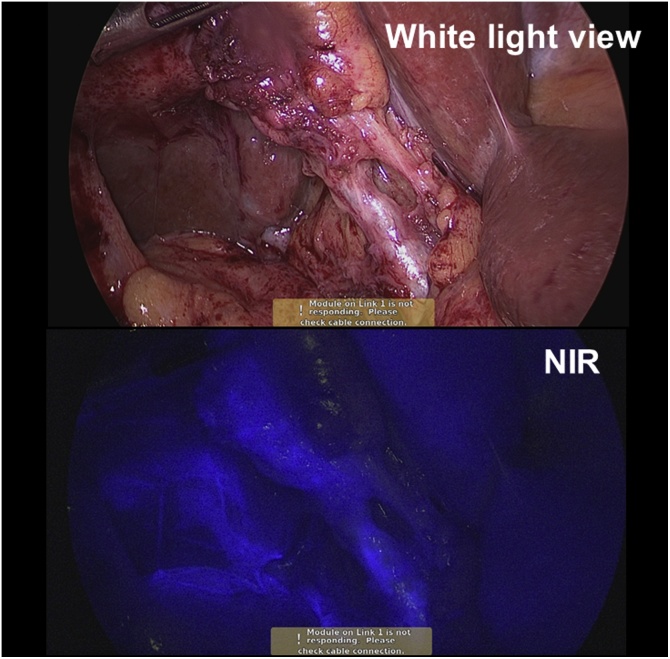
Angulated cystobiliary junction of case 2. There is additional enhancement in pericystic region under NIRF but no bile-content material seen under white light.

During surgery, dense fibrotic changes and scarring was observed in the Calot’s triangle. In case 2, there was dense desmoplastic omental adhesion around the gallbladder. Adhesiolysis was done to expose the gallbladder. The cystic duct and common bile duct was clearly identified under the guidance of NIRF cholangiography. Angulated cystobiliary junctions were identified in both cases (Fig. 2, Fig. 3). After successfully achieved CVS, an additional 1 ml (0.025 mg/ml) of ICG was administered intravenously for differentiating the cystic artery.

An additional fluorescent enhancement other than biliary system was observed in pericystic region, which was identified negative for bile leakage under white light view (Fig. 3). ICG contamination through the lymphatic drainage secondary to the inflammatory process was considered.

The total operation time for cases 1 and 2 were 84 and 125 min, respectively. Both surgeries were completed without any intra or post-operative complications. The follow-up was uneventful.

## 3 Discussion

The study showed trans-PTGBD ICG injection would reduce the noise-to-signal ratio by reducing liver fluorescence and thus increasing the contrast with higher biliary fluorescence signal, but it may not decrease operation time as it does not change the inflammatory and fibrotic status. For LC, the total intraoperative time is not a reliable parameter to assess the utility of NIRF technique as the total time can be influenced by the pericystic complications like severe inflammation, adhesions, and fibrotic tissue. Comparing with case 1, case 2 presented the pericystic tissue that was more fibrotic. All factors indicated a more severe inflammation and fibrosis, which explains the extended operation time in case 2. The mean operation time for LC with NIRF via IV injection was reported 75–90 min in the previous studies [[14], [15], [16]]. In terms of operating time, thus, the trans-PTGBD ICG administration in these two cases performed similar to IV ICG administered cases in other studies.

The time interval and the dosage of ICG for IV administration is yet to optimize, but it goes without saying that intravenous administration tends to have high noise-to-signal ratio because of prolonged hepatic fluorescence. While direct intracystic ICG injection might offer better solution to lower this noise-to-signal ratio by avoiding direct hepatic fluorescence, it might lead to major contamination of the surgical field as reported by Liu et al. [10]. Five out of 28 cases in this study that received direct intracystic injection with purse-string suture resulted in ICG leakage. Once ICG leaks into the abdominal cavity, it binds with proteins and emits strong signals [[17], [18], [19]] that occults critical structures of cholecystectomy. Intraoperative ICG spillage can cause fluorescent stain under NIR view that cannot be cleared immediately with suction or gauze mopping, which can obscure our interpretation to the fluorescent signal. To prevent from spills and minimize the possibility of ICG contamination to the extrabiliary peritoneum around the surgical field, intracystic ICG administration through a mature fistularized drain tubing provides a convenient and natural route for patients initially treated with PTGBD.

Though there was an additional fluorescent enhancement in pericystic region in case 2, there was no evident bile-content material under white light to suggest bile leakage. Lymph spillage could have occurred during gallbladder dissection. ICG may enter the lymphatic system through the necrotic gallbladder mucosa into the submucosal lymphatic drainage and the associated adhesive tissue. Despite the minor contamination, it did not interfere the identification of extrahepatic biliary structures in this study. Yet, serious contamination would obscure the surgical view.

NIRF provides an easier and safer option to visualize extra-hepatic biliary tree intraoperatively as compared to intraoperative cholangiography as it is radiation-free and non-invasive technique with real-time visualization [4]. However, in one study [20] evaluating multimodal gallbladder surgery navigation, Diana et al. compared the overall satisfaction where NIRF cholangiography was reported with a worse satisfactory result because of high noise-to-signal ratio, compared to intraoperative cholangiography and intraoperative augmented reality. Administering ICG via intracystic injection would profoundly reduce liver enhancement and overcome this issue and increase surgeon’s satisfaction with NIRF cholangiography.

## 4 Conclusion

In conclusion, intracystic injection of ICG reduces noise-to-signal ratio in comparison to IV injection by only illuminating the extra-hepatic biliary system. It could not reduce operation time, but could increase surgeon’s satisfaction with NIRF cholangiography. ICG spillage through lymphatic system may occur during gallbladder dissection in significant pericystic inflammatory adhesion cases which further obscures the florescent view. However, the ICG lymphatic fluorescent noise did not interfere with extrabiliary identification.

## Funding

This research did not receive any specific grant from funding agencies in the public, commercial, or not-for-profit sectors.

## Ethical approval

This study was approved by the institutional review board IRB of Show Chwan Memorial Hospital, Taiwan approval no. 1070507. The requirement of informed consent was waived for the study.

## Consent

Written informed consent was obtained from the patients for publication of this case report and the accompanying images. All identifying details have been omitted. A copy of the written consent is available for review by the Editor-in-Chief of this journal on request.

## Author contribution

Man-Ling Jao, Hon Phin Wong: Data Curation, Investigation, Writing-Original draft.

Yen-Yu Wang and Kai-Che Liu: Conceptualization, Supervision, Project Administration.

Sayali Bachhav: Writing-Original draft, Writing-Review and Editing.

All authors have contributed equally in the final manuscript approval and otherwise.

## Registration of research studies

Name of the registry: ResearchRegistry.com.

Unique Identifying Number (UIN): researchregistry5213.

## Guarantor

Dr. Hon Phin Wong.

Dr. Kai-Che Liu.

## Provenance and peer review

Not commissioned, externally peer-reviewed.

## Declaration of Competing Interest

All authors have no conflicts of interests or financial ties to disclose.
